# Ferroptosis and oxidative stress in endometriosis: A systematic review of the literature

**DOI:** 10.1097/MD.0000000000037421

**Published:** 2024-03-15

**Authors:** Chenghong Ni, Dingheng Li

**Affiliations:** a Department of Hangzhou Normal University, Hangzhou, Zhejiang Province, China; b Department of Obstetrics and Gynecology, Hangzhou Women’s Hospital, Hangzhou, Zhejiang Province, China.

**Keywords:** endometriosis, ferroptosis, iron metabolism, oxidative stress

## Abstract

**Background:**

Endometriosis (EMT) a common gynecological condition in women, an inflammatory disease characterized by the presence of endometrial tissue on organs and tissues in the pelvis, and is mainly associated with chronic pelvic pain and infertility. As the etiology has not been fully elucidated, current treatment is limited to surgery, hormones and painkillers, with more side effects and difficulty in achieving long-term relief. Oxidative stress manifests itself as an overproduction of reactive oxygen species, which has an integral impact in the pathology of female reproductive disorders. In this review, we evaluate the mechanisms of iron overload-induced oxidative stress and ferroptosis in EMT and their pathophysiological implications.

**Methods:**

Because the etiology has not been fully elucidated, current treatments are limited to surgery, hormones, and painkillers, which have many side effects and are difficult to achieve long-term relief.

**Results:**

We interpreted that antioxidants as well as ferroptosis inducers show promising results in the treatment of EMT, but their application in this population needs to be further investigated.

**Conclusion:**

In combination with the interpretation of previous studies, it was shown that iron overload is present in the peritoneal fluid, endometriotic lesions, peritoneum and macrophages in the abdominal cavity. However, the programmed cellular ferroptosis associated with iron overload is resisted by endometriotic foci, which is critical to the pathophysiology of EMT with local iron overload and inflammation.

## 1. Introduction

Endometriosis (EMT) is a common chronic inflammatory disease in women, which refers to the presence of endometrial glands and mesenchyme outside of the uterine cavity, with common sites including the fallopian tubes, ovaries, bladder, rectum, and uterine myometrium.^[1]^ EMT can be categorized into three subtypes based on histopathology and anatomical location: superficial peritoneal endometriosis, ovarian endometriosis, and deep infiltrating endometriosis.^[2]^ As EMT is usually associated with fibrosis and adhesions, the main clinical manifestations contain abdominal pain, chronic pelvic pain, which is aggravated during sexual intercourse, urination, and defecation.^[3]^ No definitive treatment exists for EMT, which as an estrogen-dependent disease,^[4]^ can reduce pain and slow disease progression by decreasing estrogen production and suppressing ovulation. Surgical treatment by complete excision of deep lesions is one of the effective means for female patients with deep infiltrating endometriosis who fail to respond to pharmacologic treatment.^[5]^

There are a few theories about the origin of EMT including retrograde menstruation, corpora cavernosa chemotaxis, lymphatic and vascular spread, hormones, immune factors and extrauterine stem cell differentiation.^[6]^ The most recognized and plausible of these is the “retrograde menstruation” proposed by J.A. Sampson in 1927,^[7,8]^ which is the retrograde transport of erythrocytes and endometrial tissue from menstrual blood through the fallopian tubes, which are subsequently implanted in the peritoneum causing inflammation, immune dysfunction, and oxidative stress. Among them, oxidative stress is the imbalance between reactive oxygen species and antioxidants, which is the main cause of cell death including apoptosis, autophagy and ferroptosis.^[9,10]^ Ferroptosis is a novel mode of cell death mediated by iron, which manifests itself in the form of cytoarchitectural disruption caused by lipid peroxidation.^[11]^ Ferroptosis is closely related to the pathogenesis of cancer, ischemia-reperfusion injury or ischemic organ damage, neurodegenerative diseases, stroke, and renal failure.^[12]^ The exact mechanism of EMT is currently unknown, but recent studies suggest that EMT is thought to be closely related to iron-dependent oxidative stress-mediated ferroptosis.^[13–15]^

The aim of this review is to elucidate the formation of excess iron in endometriotic lesions and the oxidative stress it induces, the mechanism of ferroptosis and the resistance of EMT to ferroptosis, and new therapeutic options for endometrial hyperplasia-associated oxidative stress and ferroptosis.

## 2. Excess iron in EMT causes oxidative stress

Most of the iron in the body is found in circulating erythrocytes, and the role of iron includes oxygen transport, metabolic reactions and DNA synthesis.^[16]^ At the macroscopic level iron deficiency leads to cartilage dysfunction, cognitive developmental deficits, reduced organic capacity and adverse pregnancy outcomes. Excessive accumulation of iron like damages multiple organs including parenchymal organs such as kidney, heart and liver causing iron loading diseases.^[17]^ Studies have shown that for patients with EMT higher levels of iron, ferritin, transferrin, hemoglobin are present in the abdominal fluid, ovarian EMT cysts and follicles.^[18,19]^ This phenomenon may be related to increased erythrocyte degradation and influx caused by menstrual reflux and repeated bleeding from endometriotic cysts.^[20]^ For the refluxed endometrial tissue as well as erythrocytes will be phagocytosed, absorbed and degraded by macrophages and exist in the form of iron-containing hemosiderin. Lambrinoudaki et al^[21]^ suggested that high levels of iron cannot be metabolized in a timely manner leading to the abnormal activation of macrophages, which are abnormally activated to phagocytose haptopodophyllin and erythrocyte complexes through the CD163 receptor, and at the same time, release hemoglobin into the peritoneal fluid, which then metabolizes hemoglobin via heme oxygenase-1 to generate reactive iron as well as iron-ferritin deposits.^[22]^ This prevents timely removal by the iron homeostasis and iron scavenging system systems, ultimately leading to an environment of iron overload in EMT peritoneal fluid and ectopic lesions.^[19]^

Erythrocyte-derived iron is one of the inducers of oxidative stress, which affects the redox state in cells and tissues, and excess iron catalyzes the Fenton reaction (Fe^2+^ + H_2_O_2_ → Fe^3+^ + OH^-^ +^.^OH),^[23]^ which produces an excess of reactive oxygen species (ROS, the main types of which: superoxide anion, hydrogen peroxide, and hydroxyl),^[24]^ and the targets of ROS include lipids, proteins, and nucleic acids, and highly reactive hydroxyl radicals can alter cellular structure and function, and produce cytotoxic effects on living cells.^[25]^ ROS due to iron overload activate ectopic endometrial cell proliferation, angiogenesis and adhesion. Animal models have been shown that mice using iron agents produce more diseased cells to promote EMT growth than controls.^[26]^ Overall, high iron levels in EMT have an effect on lesions through oxidative stress mediated by free iron in the Fenton reaction.

## 3. Ferroptosis

### 3.1. Mechanism of ferroptosis

**Figure 1.**
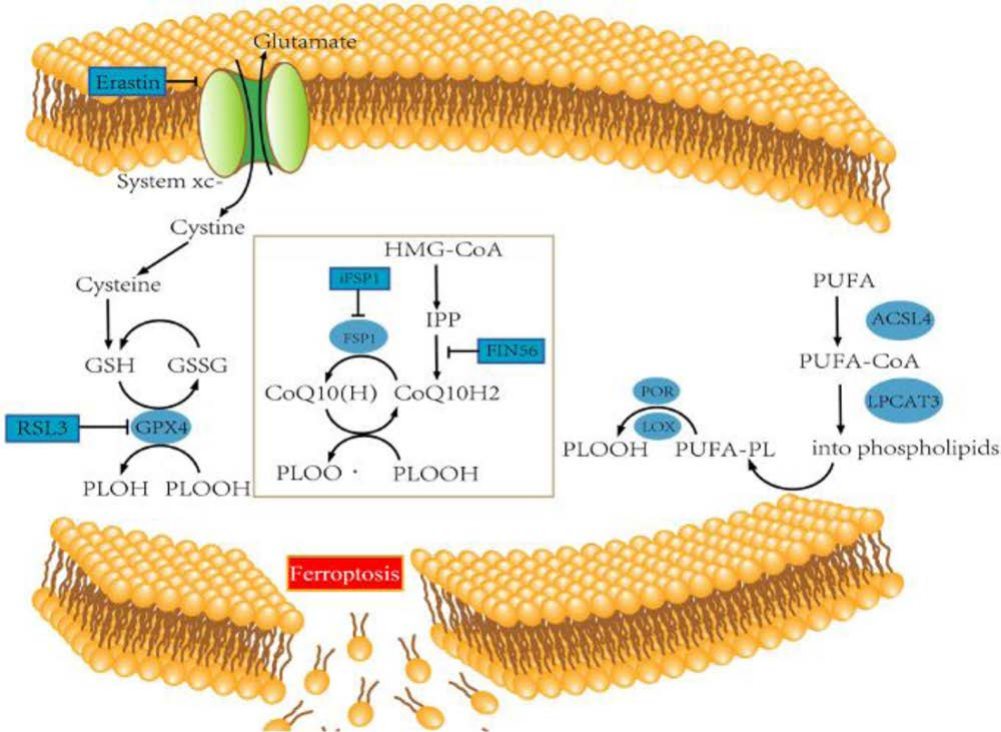
Ferroptosis inhibition pathway and phospholipid peroxidation. Where ferroptosis regulators are marked with blue boxes. Promotion and inhibition are indicated by arrows and short horizontal lines, respectively. GSH/GPX4 system takes up cystine and forms GSH as a cofactor for GPX4. GOPX4 converts PLOOH to PLOH. COQ10/FSP1 system in which FSP1 reduces ubiquinone to ubiquinol to terminate lipid peroxidation. ACSL4 and LPCAT3 esterify PUFA into phospholipids to increase long-chain PUFA, and LOX and POR catalyze PUFA-PL to form PLOOH. ACSL4 = Acyl-CoA synthetase long-chain family member 4, CoQ10 = coenzyme Q10, FSP1 = ferroptosis suppressor protein 1, GPX4 = glutathione peroxidase 4, GSH = glutathione, LOX = lipoxygenases, LPCAT3 = Lys phosphatidylcholine acyltransferase 3, POR = p450 oxidoreductase, PUFA-PL = Phospholipids Containing Polyunsaturated Fatty Acids.

Ferroptosis as a novel cell death mode was firstly proposed by Dixon et al^[10]^ in 2012, which is mainly manifested as cell membrane damage caused by lipid peroxidation, and iron overload is the main causative factor of this regulated cell death. Lipid peroxidation targets polyunsaturated fatty acids (PUFA) in cell membranes, and Acyl-CoA Synthetase Long-chain Family Member 4 can bind PUFA to coenzyme A to form acyl coenzyme A esters and re-esterify into phospholipids via Recombinant Lysophosphatidylcholine Acyltransferase 3 to form Phospholipids Containing Polyunsaturated Fatty Acids (PUFA-PL).^[27]^ The phospholipid radicals generated in PUFA-PL react with molecular oxygen to form phospholipid peroxyl radicals, which then combine with hydrogen from another PUFA to form PLOOH, and these large amounts of secondary products can ultimately lead to damage to organelles or cell membranes (refer to Fig. 1).^[28,29]^ The ferroptosis pathway is affected by a variety of enzymes, such as certain lipoxygenases that can directly oxidize PUFA and PUFA-containing lipids in biological membranes, thereby increasing the likelihood of ferroptosis.^[30]^ Cytochrome p450 oxidoreductase affects iron metabolism by removing hydrogen from PUFA or by electron transfer through reduction of trivalent iron (Fe3+) to its ferrous form (Fe2+).^[31]^ Lipoxygenases Isoforms and p450 oxidoreductase can catalyze the production of PLOOH from PUFA-PL to induce ferroptosis.^[32]^ Overall, the molecular mechanisms of ferroptosis are regulated by cellular signaling pathways as well as genes, but are mediated in two main ways, transporter protein-dependent pathways (e.g., decreased cysteine or glutamine uptake and increased iron uptake) and enzyme-regulated pathways (e.g., inhibition of the glutathione (GSH) peroxidase 4 (GPX4) antioxidant system).

For this mechanism of ROS-mediated death has received extensive attention in the last few years, such as induction of ferroptosis to fight cancer cells, and inhibition of ferroptosis for neuroprotection.^[33]^ Ferroptosis has been demonstrated in EMT, where several studies have shown that endometrial cells are not destroyed in an environment of excess iron, but rather survive, implant, and grow in ectopic lesions, suggesting that resistance to ferroptosis is critical for EMT survival.^[14]^

### 3.2. Pathways affecting ferroptosis

#### 3.2.1. Effect of GSH and cysteine GPX4 on ferroptosis.

The system xc-antiporter retrogrades intracellular glutamate with extracellular cysteine,^[34]^ which is the precursor amino acid for the synthesis of GSH, which is the main intracellular antioxidant, and a cofactor for the normal function of GPX4. GPX4 is a selenoprotein that acts as a phospholipid hydroperoxidase converting (PLOOH) to the corresponding alcohol (PLOH) to achieve antioxidant effects.^[35]^ The first system xc-transporter consists of a heterodimeric protein complex composed of light chain subunit solute carrier family 7 member 11 (SLC7A11) and heavy chain subunit solute carrier family 3 member 2, through which cysteine enters into the cell, and due to the limited concentration of cysteine in the cell, cysteine is considered to be the rate-limiting precursor of GSH synthesis. rate-limiting precursor for synthesis.^[36]^ Secondly GSH is a tripeptide composed of glutamate, cysteine and glycine and is synthesized through glutamate-cysteine ligase (GCL), therefore GCL determines the efficiency of GSH synthesis.^[37]^ The final GPX4 is a key element in the antioxidant system and can directly reduce lipid hydroperoxides in membranes to nontoxic lipid alcohols compared to other isoenzyme members of the GPXs family, that is, GPX4 reduces cell death due to lipid peroxidation induced by the accumulation of ROS (refer to Fig. 1).^[38,39]^ Expression of GPX4 is regulated by GSH and selenium, and its catalytic requirement is for selenium substituted cysteine and two electrons provided by GSH to accomplish this, and GPX4 is inactivated upon GSH depletion.^[40]^ Thus, maintaining GSH synthesis or GPX4 activity protects cells from death triggered by various oxidative stress conditions.

The regulation of various antioxidant inhibitors as well as ferroptosis inducers has been implicated throughout the ferroptosis process. For example, studies have reported that the tumor suppressor-related genes BRCA1, p53, and ARF inhibit SLC7A11 activity,^[41,42]^ and similarly tumors can up-regulate SLC7A11 expression and thus affect ferroptosis by enhancing ETS proto-oncogene 1 and transcription factor 4.^[43]^ Three isoforms of GPX4 exist: mitochondrial, cytoplasmic and nuclear, and exactly which organelle serves as an independent regulator of localized lipid peroxidation is currently unknown. Based on its unique function, GPX4 is considered to be a potent inhibitor of ferroptosis and plays a key role in regulating ferroptosis.^[44,45]^ It has been reported that GPX4 is commonly expressed in cancer cells to manipulate ferroptosis sensitivity, and the compound RSL3, which promotes ferroptosis, directly inactivates GPX4, while another compound, erastin, inactivates GPX4 by inhibiting cytosolic cystine input.^[38,46]^ In addition, Buthionine sulfoximine can induce ferroptosis or enhance cellular sensitivity to ferroptosis induced by other drugs by inhibiting GCL.^[47]^

Current studies have demonstrated that endometrial surface and glandular epithelial GPX expression in patients with EMT changes in a phase-dependent manner during the menstrual cycle. Moreover, some proteins of the GPX family are abnormally expressed in follicular fluid in response to the female menstrual cycle, with the highest concentration of GPX in rat ovarian and human follicular fluid during ovulation.^[48,49]^ In addition, EMT shows elevated levels of GSH, suggesting the presence of prior oxidation-related alterations. An animal study found that iron overload in the peritoneal fluid of endometriotic lesions in mice induced ferroptosis, impaired oocyte maturation, and disrupted blastocyst formation, leading to infertility.^[50]^ In summary, endometrial cells rely on a GPX-dependent antioxidant system to gain intrinsic resistance to ferroptosis; however, current studies have not yet elucidated the molecular mechanisms underlying elevated GPX in EMT and the effects of specific GPX4 on EMT.

#### 3.2.2. Effect of Ferroptosis suppressor protein 1 on ferroptosis.

Ferroptosis suppressor protein 1 (FSP1) was originally defined as a pro-apoptotic gene11 that protects against ferroptosis induced by GPX4 deletion.^[51]^ Doll et al^[52]^ screened for genes complementing GPX4 deletion and found that overexpression of FSP1 resisted ferroptosis induced by GPX4 inhibition. The current study demonstrated that the inhibition of ferroptosis by FSP1 is mediated by ubiquinone (also known as coenzyme Q10, CoQ10), a reduced form of ubiquinol that recycles lipid peroxidation radicals to promote lipid peroxidation, and that FSP1 can catalyze the regeneration of CoQ10 via NAD(P)H.^[53]^ The FSP1-CoQ10-NAD(P)H pathway can be act as a pathway independent of GPX4 and GSH to synergistically inhibit phospholipid peroxidation and ferroptosis.^[54]^

In the FSP1-CoQ10-NAD(P)H pathway, a variety of enzymes and inducers can affect ferroptosis efficiency. For example, coenzyme Q2 (CoQ2) catalyzes CoQ10 biosynthesis, and over depletion of CoQ2 inhibits the anti-ferroptosis function of FSP1 and ferroptosis in cells treated with the CoQ2 inhibitor, 4-chlorobenzoic acid was more pronounced.^[55]^ Idebenone, a soluble analog of CoQ10, was effective in preventing lipid peroxidation from affecting ferroptosis. Tetrahydrobiopterin/dihydrobiopterin, an intermediate in the synthesis and metabolism of GTP cyclohydrolase 1, can regulate CoQ10 to inhibit cellular ferroptosis.^[53]^ A metabolic pathway for the synthesis of isopentenyl pyrophosphate and dimethylallyl pyrophosphate, which includes cholesterol, CoQ10, and isopentenyl pyrophosphate, is known as The Mevalonate Pathway, from acetyl coenzyme A.^[56,57]^ Isopentenyl pyrophosphate is the precursor of CoQ10 and can regulate CoQ10 to inhibit cellular ferroptosis (refer to Fig. 1).^[53]^ precursor of CoQ10 can act as an endogenous lipophilic antioxidant to protect cells from ferroptosis.^[58]^ In addition, cholesterol has a corresponding sensitivity to free radicals such as hydroxyl radicals, and excess exogenous cholesterol peroxides can induce cell death,^[59]^ GPX4 is the only known enzyme capable of directly scavenging cholesterol peroxides, and cells overexpressing GPX4 are extremely resistant to cholesterol hydroperoxide-induced oxidative cell death.^[60]^ In addition, Nakamura et al^[61]^ screened compounds of 3-phenylquinazolinones (represented by icFSP1) as potent FSP1 inhibitors. icFSP1 does not competitively inhibit FSP1 enzyme activity but rather triggers the release of FSP1 from the membranes and prior to induction of ferroptosis. Subcellular relocalization of FSP1 condensation to inhibit ferroptosis.

Relatively little research has been done on FSP1 in EMT, and one clinical study found that the proportion of CD31 and FSP1 double-positive cells in ovarian EMT lesions was 74.7% (±5.4%), compared with zero in normal endometrium.^[62]^ Statins can target COQ2 in the CoQ10/FSP1 axis to impede CoQ10 synthesis. An animal study using simvastatin reduced the volume of active lesions in EMT.^[63]^ In addition, the use of statins affects the activity of enzymes in the mevalonate pathway, which affects the efficient translation of GPX4 and thus sensitizes cells to ferroptosis, thus making cholesterol a modulator of lipid peroxidation and ferroptosis. For the current study both from the pathogenesis point of view and the expression of FSP1 in EMT need to be further explored.

## 4. Ferroptosis in EMT

### 4.1. Effect of ferroptosis in EMT

The first thing we can confirm is that iron overload in endometriotic stromal cells induces ferroptosis. However, current studies on ferroptosis in EMT have found that the role of ferroptosis in EMT appears to be bidirectional. One study found that ferroptosis in endometriotic cells triggers cytokines and activates downstream regulatory pathways to promote proliferation and angiogenesis in surrounding tissues.^[64]^ In addition, Li et al^[65]^ found that ferroptosis in ovarian endometriotic mesenchymal stromal cells promoted the upregulation of vascular endothelial growth factor A (VEGFA) and interleukin-8 (IL-8) in ectopic lesions. These pro-inflammatory and angiogenic cytokines promote cell proliferation, adhesion, and angiogenesis in ectopic endometrial tissues, thereby promoting the development of EMT.^[66]^ In addition, iron-dependent ferroptosis can promote the development of EMT by impairing phagocytosis in macrophages and by upregulating the expression of interleukin-8 and vascular endothelial growth factor-A in macrophage THP-1 cell lines.^[67]^ Recent studies have found that women with EMT have a more M2 macrophage phenotype in their peritoneal fluid, which promotes the proliferation and growth of ectopic endometrial tissues by providing better resistance to ferroptosis and releasing anti-inflammatory cytokines, growth factors, and other reparative components compared to M1 macrophages M2 macrophages.^[68,69]^

In fertile women with EMT, it has been found that iron overload in follicular fluid and granulosa cells induces ferroptosis, impairs oocyte maturation, and disrupts blastocyst formation, leading to infertility.^[50]^ A mouse study found that exposure of mouse embryos to EMT peritoneal fluid impaired early embryonic development and caused embryotoxicity, increasing adverse pregnancy outcomes.^[70]^ In addition, mouse oocytes that were also exposed to peritoneal fluid from women with EMT developed ferroptosis caused by iron overload.^[71]^ For the ferroptosis inhibitor ferritin-1 showed some protective effects on fertility.^[72]^ ferroptosis affects oocyte and blastocyst development, which has been linked to infertility in patients with EMT.

### 4.2. Mechanisms of ferroptosis resistance in EMT

A growing body of evidence validates that dysregulation of iron homeostasis and resistance to ferroptosis promote the value-addition and implantation of ectopic lesions.^[73–75]^ First, the dysregulation of iron homeostasis manifests itself as iron overload, and Mori et al^[76]^ studied the first catalytic (unstable) Fe (II) with the RhoNox-1 fluorescent probe to find that the catalytic Fe(II) content in ectopic endometrial stromal cells was higher than that in normal in situ endometrial stromal cells. Ectopic endometrial stromal cells express higher levels of transferrin receptor, which leads to sustained iron uptake. In addition, ectopic endometrial stromal cells show higher iron storage capacity.^[77]^ High levels of iron promote oxidative stress as well as inflammatory responses, as evidenced by increased erythrocyte counts, elevated transferrin expression, and elevated levels of polyunsaturated fatty acid (PUFA) phospholipids^[78]^ and enable ectopic endometrial tissues to increase their cell viability and energy metabolism through certain pathways, protecting the cells from iron-mediated cytotoxicity.^[79,80]^ Examples include the resynthesis of haptoglobin in endometriotic lesions and a significant increase in the expression of the heme oxygenase 1 gene, both of which have important antioxidant and immunomodulatory roles, and are primarily involved in stimulus responses, the immune system, metabolism, localization and cellular processes.^[81,82]^

The resistance of EMT to ferroptosis is demonstrated by the expression of multiple genes to regulate lipid peroxidation and amino acid metabolism to influence ferroptosis in ectopic lesions. Experimental evidence suggests that long chain non-coding ribonucleic acid (RNA), which are RNA molecules more than 200 nucleotides in length and do not have protein coding capacity, play an important regulatory role in the pathophysiologic mechanisms of EMT.^[83,84]^ Expression of the long chain non-coding RNA ADAMTS9-AS1 was significantly upregulated in ectopic endometrium, and knockdown of ADAMTS9-AS1 reduced cell viability and migration.^[85]^ ADAMTS9-AS1 could inhibit GPX4 in ectopic endometrium by sponging miR-6516-5p to inhibit the mRNA and protein expression levels of GPX4, a key inhibitor of ferroptosis, thus acting as a competing endogenous RNA,^[86]^ that is, a mechanism by which ectopic endometriotic cells gain resistance to ferroptosis by upregulating GPx4 and its upstream regulatory target GSH [10927050]. Long noncoding RNA metastasis-associated lung adenocarcinoma transcript 1 (MALAT1) promotes oxidative stress associated with reactive oxygen species production in many diseases.^[87]^ MALAT1 regulates the expression of MUC1 (mucin 1), an inhibitor of ferroptosis, by competing with miR-145-5p for endogenous RNA, and miR-145-5p inhibition-mediated ferroptosis can be eliminated by knockdown of MUC1.^[88,89]^ By knocking down MALAT1 in a mouse model contributed to the promotion of erastin-induced ferroptosis in endometriotic cells through miR-145-5p/MUC1 signaling.^[15]^

In addition, high cholesterol is correlated with ferroptosis in women with EMT, and it has been found that women with EMT usually have an abnormal lipid profile with high levels of oxidized low-density lipoprotein (LDL) and higher concentrations of oxidized low-density lipoprotein and PUFA-phospholipids in patients with severe lesions.^[90,91]^ The molecular mechanisms underlying the association between EMT and high cholesterol are unclear, and possible explanations are high levels of cholesterol activity and oxidized metabolites of cholesterol in the peritoneal cavity suggesting that the mevalonate pathway is abnormally active in patients with EMT.^[92,93]^ One of the mevalonate products, CoQ10, acts as an endogenous antioxidant that protects endometriotic cells against ferroptosis. A prospective cohort study showed that laparoscopically confirmed EMT was associated with an increased lifetime risk of developing hypercholesterolemia and hypertension, and that the presence of hypercholesterolemia and hypertension increased the risk of a subsequent diagnosis of EMT.^[94]^ Thus, hyperactivation of the mevalonate pathway in women with elevated cholesterol may play a major role in protecting endometriotic cells from ferroptosis.

Finally, fibulin 1 has been reported to play a role in resistance to ferroptosis in EMT.^[95]^ Fibulin 1 is a glycoprotein that maintains the stability of the extracellular matrix and is a tumor suppressor in gastric, prostate, breast, and ovarian cancers.^[96]^ Fibronectin-1 was found to be overexpressed and inhibited ferroptosis in endometrial ectopic stromal cells. The inhibition of ferroptosis promoted the proliferation and migration of endometriotic stromal cells.^[97]^

In summary, several mechanisms for the regulation of ferroptosis in EMT have been proposed, and aberrant resistance to ferroptosis in EMT is a key factor in lesion establishment and growth.

## 5. Antioxidant as well as the possibility of targeting ferroptosis for the treatment of EMT

In clinical practice most patients with EMT present with unrelieved pelvic pain and infertility, but some patients have no clinical manifestations.^[98,99]^ The prevalence of EMT in women with pelvic pain is approximately 30% to 45% of the infertile population.^[100]^ Previous studies have shown that neurogenic inflammation, macrophages, lipid peroxidation and prostaglandins play a crucial role in the pathophysiology of endometriotic pain.^[101,102]^ However, the understanding of the underlying mechanisms of the disease is still limited, and the treatment of patients is divided into pharmacological treatments such as nonsteroidal anti-inflammatory drugs, contraceptives, progestins, progesterone receptor antagonists, danazol, and gonadotropin-releasing hormone agonists to reduce estrogen levels or relieve pelvic pain.^[103,104]^ These usual have little effect or side effects such as hepatic impairment, metabolic disturbances and long dosing times. Comparatively another treatment modality: laparoscopic surgical treatment, as an invasive procedure, is not the most effective means as it is effective in improving pain symptoms and fertility,^[105]^ but some patients also require second and third surgeries after surgery.^[106,107]^

This summary summarizes the role of antioxidant as well as ferroptosis-specific treatment modalities on the improvement of EMT symptoms and disease prognosis, providing possibilities for future clinical treatment.

### 5.1. Antioxidants in EMT

Oxidative stress plays a crucial role in the pathogenesis of EMT by correcting the oxidative stress caused by the imbalance between reactive oxygen species and antioxidants, which reduces disease symptoms, tissue destruction, and proliferation and activity of endometriotic cells.^[108]^ Therefore, novel therapeutic approaches for this disease could start from targeting oxidative stress, such as antioxidant supplementation may be effective in treating and reducing the severity of EMT. Moreover, the current studies on oxidative stress markers including serum heat shock protein, lipid levels (triglycerides, total cholesterol, and LDL), malondialdehyde (MDA), and vitamin E,^[109–111]^ which are useful for subsequent dynamic monitoring of antioxidant effects of antioxidants. Some of the antioxidants that have been studied to stop oxidative stress in EMT are discussed below.

#### 5.1.1. Effect of vitamins C and E on EMT.

Vitamin C is a water-soluble vitamin that acts as a physiological antioxidant to reduce the cellular damage caused by oxidative stress.^[112,113]^ Vitamin E is a fat-soluble antioxidant that inhibits lipid peroxidation and inflammation and prevents diseases caused by oxidative stress.^[114,115]^ Both vitamin C and vitamin E have well-established antioxidant properties and can be considered to neutralize free radicals and reactive oxygen species produced by endometriotic cells.^[116]^ More studies have been conducted to describe the effects of vitamins C and E on EMT, which can be supplemented to reduce oxidative stress markers and pain.^[117]^ Amini et al performed a study of laparoscopically confirmed EMT with placebo tablets (control group) or a combined supplement of vitamins C (1000 mg) and E (800 IU) taken as a daily placebo for 8 weeks. After 8 weeks of treatment compared to the placebo group, pelvic pain, dysmenorrhea and pain severity were significantly reduced, MDA and ROS were significantly reduced, however, there was no significant decrease in total antioxidant capacity.^[118]^ In a recent study, Lu et al^[119]^ stated that oral vitamin C (1000 mg/d) for 2 months did not affect OS markers (ROS, TAC, SOD, and MDA) in patients with EMS. Erten et al investigated the effect of vitamin C on the prevention and regression of endometriotic implants in a rat model of EMT. The area and volume of lesion implants were smaller in mice that received 500 mg/kg of vitamin C via intravenous infusion every 2 days compared to the control group, suggesting that vitamin C may have an inhibitory effect on the prevention of endometriotic implants and regression of endometriotic implant volume.^[120]^

#### 5.1.2. Effect of N-acetylcysteine on EMT.

N-acetylcysteine (NAC) is the acetylated form of the amino acid cysteine, a synthetic derivative of the endogenous amino acid L-cysteine and a precursor of GSH and is often used as the sole therapeutic option for patients suffering from acetaminophen overdose.^[121]^ not only does NAC regulate oxidative stress, but it also influences apoptosis and inflammation, and the nucleophilic free sulfhydryl group of NAC makes it able to act as a direct antioxidant against many electrophilic oxidizing radicals.^[122,123]^ The therapeutic activity of NAC relies on its ability to provide a reduced sulfhydryl fraction and act as a precursor to GSH to replenish GSH required for ferroptosis, and in EMT it may possess some therapeutic effect.^[124]^ Pittaluga et al^[125]^ mouse model experiments demonstrated that NAC could alter cell behavior by switching cells from a proliferative state to a differentiated state, contributing to a reduction in endometrial lesion size, tissue inflammation, and cellular invasion. Porpora et al^[126]^ conducted an observational cohort study in which NAC 600 mg was administered orally to patients with ovarian EMT three times a day for three consecutive days a week for three months and in NAC-treated patients, the mean cyst diameter was slightly reduced (−1.5 mm), while it was significantly increased (+6.6 mm) in untreated patients. Thus, NAC represents a simple and effective treatment for EMT with no side effects and is a suitable method for women wishing to become pregnant.

#### 5.1.3. Effects of melatonin on EMT.

Melatonin (N-acetyl-5-methoxytryptamine) is a hormone synthesized and released by the pineal gland at night that regulates reproductive function by regulating the body’s circadian rhythms, development as well as directly acting on the hypothalamic-pituitary-ovarian axis.^[127]^ Melatonin has good antioxidant properties, acting as a free radical scavenger of hydrogen peroxide and nitric oxide, reducing lipid peroxidation and ferroptosis markers.^[128,129]^ Other biological functions of melatonin include regulation of steroid hormone production, promotion of apoptosis, modulation of immunity and trophic neurological effects,^[130,131]^ and modulation in cardiovascular, endocrine, immune and central nervous systems.^[132,133]^ Similarly, melatonin has been shown to play a key role in EMT, with a case-control study showing a 50% increased risk of EMT in night shift work, suggesting a possible correlation between melatonin and EMT.^[134]^ Studies using animal models of EMT have shown that melatonin reduces the size and weight of cysts and inhibits the proliferation and implantation of lesions.^[135,136]^ Melatonin may prevent or abrogate EMT by regulating metalloproteinase (MMP) gene expression and activity, and MMP has been shown to play a crucial role in the pathogenesis of EMT, with elevated levels of MMP2, MMP3, and MMP9 expression in ectopic endometrium and EMT cells.^[137]^ Additionally, melatonin has been found to possess analgesic properties that can alleviate inflammatory chronic pelvic pain, and a double-blind, placebo-controlled clinical trial demonstrated that melatonin treatment reduced daily pain scores by 80.95% compared to placebo.^[138]^ While traditional analgesics can cause side effects such as gastric ulcers with long-term use, current evidence suggests that short-term use of melatonin supplements is safe and has been shown to be safe for use in obstetrics and reproduction-related disorders, with no negative effects on the reproductive system.^[139]^

### 5.2. Ferroptosis inducers in EMT

Sensitivity to ferroptosis is influenced by factors related by iron, GSH/GPx4, mevalonate and polyunsaturated fatty acids. Therapeutic strategies targeting ferroptosis hold great promise in preclinical studies of various pathological processes such as cancer, neurodegenerative diseases and cardiovascular diseases. Two classes of ferroptosis modulators have been studied: activators of novel anticancer agents and inhibitors of ferroptosis as novel cytoprotective agents.^[140]^ Compounds that induce ferroptosis include erastin, imidazolidinone erastin (IKE), salazosulfapyridine and sorafenib.^[141,142]^ They induce ferroptosis by blocking the uptake of SLC7A11 for cystine in the system xc- antiporter, of which erastin, IKE, luzosulfapyridine and sorafenib are collectively referred to as class 1 ferroptosis inducers (FIN). In addition, in vitro and animal studies have emphasized ferroptosis as a new therapeutic target for EMT.^[14]^ However, there is no reliable evidence to assess the therapeutic efficacy of ferroptosis inducers or inhibitors in patients with EMT. The long-term effects of ferroptosis on endometriotic cells and granulosa cells as well as oocytes due to the dual effects of ferroptosis on EMT, that is, ferroptosis inducers and inhibitors, remain unknown. Although proof-of-concept has been established in clinical studies, targeted therapies against ferroptosis are currently just beginning in EMT models.

#### 5.2.1. Ferroptosis inducer erastin.

Erastin is the most widely used small molecule inducer of ferroptosis in cellular studies and can trigger ferroptosis by inhibiting cystine/glutamate transporter receptors and causing GSH depletion and GPX4 inactivation.^[143]^ In addition, erastin can directly affect cystine uptake via voltage-dependent anion channels (VDAC).^[144]^ VDAC2 is a major channel for the entry and exit of mitochondrial metabolites, and it is an important gene in the intracellular redox reactions, and it has been demonstrated that knocking down the VDAC2 and VDAC3 leads to erastin resistance.^[145]^ Due to the poor stability of erastin as well as low in vivo solubility, it cannot be used in animal experiments, so it is not available for further clinical studies and testing. Imidazole ketone erastin is an erastin analog has high stability and can be used in animal therapy, but imidazole ketone erastin was discovered in a short period of time and has not yet been entered into clinical trials.^[44]^ A study by Li et al found that ectopic endometrial stromal cells treated with erastin significantly increased total ROS levels, lipid ROS levels, and intracellular iron levels than normal endometrial stromal cells. Moreover, condensed mitochondria were observed in endometrial ectopic stromal cells treated with erastin, and these results suggest that erastin is able to induce ectopic endometrial stromal cell death via ferroptosis. In addition, ferroprotein (a transmembrane protein is the only iron exporting protein in mammals), which is highly expressed in ectopic endometrial stromal cells, was able to resist erastin-induced ferroptosis.^[146,147]^

#### 5.2.2. Effect of statins on ferroptosis.

Statins (e.g., sorafenib) have a variety of effects including reduction of inflammatory factors, antioxidant, antiproliferative and pro-apoptotic.^[148]^ In contrast to erastin, which is still in the research phase, statins are a class of drugs that are widely used clinically for the treatment of hypercholesterolemia and dyslipidemia and are now shown to inhibit the proliferation and invasion of the endometrial stroma in vitro by blocking the mevalonate pathway,^[149]^ providing a targeted therapy for EMT. Statins as exogenous ferroptosis inducers accelerate lipid ROS accumulation and promote endometriotic cell death. In a primate model, simvastatin, a mevalonate pathway inhibitor, downregulated GPX4 expression to induce cellular ferroptosis and inhibit endometriotic cell growth.^[150]^ Statins target 3-hydroxy-3-methylglutaryl coenzyme A reductase activity in the mevalonate pathway, which is a key enzyme in the pyruvate pathway that inhibits the synthesis of CoQ10 and selenoproteins and increases the susceptibility to ferroptosis to inhibit the growth and angiogenic potential of endometriotic cells. In addition, the intermediate product of the mevalonate pathway, isopentenyl pyrophosphate, is important for the regulation of isoprenylation of Sec-tRNA, and translation of the Sec-tRNA gene regulates the activity of GPX4, which is mainly capable of scavenging lipid peroxides.^[151,152]^ Therefore, limiting GPX4 activity by inhibiting the mevalonate pathway offers a possibility for the treatment of EMT. However, granulosa cells are different from endometriotic cells, and reduced endogenous GPx4 expression in granulosa cells can further damage the cells, leading to difficulty in oocyte survival and possibly leading to infertility.^[50]^

## 6. Conclusion

This paper reviews our current understanding of the mechanisms of ferroptosis associated with EMT and discusses the research and therapeutic value of antioxidants as well as ferroptosis modifiers. Local iron overload results from erythrocyte degradation through retrograde menstruation or bleeding from ectopic endometriotic lesions, and iron overload has a direct impact on EMT lesion value-added, infertility, symptom severity, and malignancy. With recent years researchers have come to recognize and reveal the potential role of ferroptosis in EMT. They have emphasized that ectopic endometrial tissues resist iron overload-induced ferroptosis to promote ectopic lesion growth. For the diagnosis of EMT is an important issue in the long-term management of the disease, the diagnostic efficacy of imaging tests, including transvaginal ultrasound (TRUS) and magnetic resonance imaging, is not ideal, and the combination of laparoscopy and pathologic tissue biopsy is currently the gold standard for diagnosis.^[153]^ However, whether ferroptosis regulators such as GPX4, a key factor in the antioxidant system, can serve as unique markers in EMT needs to be further characterized. Second, therapeutic options for EMT can only address the various symptoms of the disease, and research targeting oxidative stress states may be the key to effective prevention of EMT. Reactive oxygen species regulate many physiological functions in reproduction, and when the balance between ROS and antioxidants is disrupted, it can lead to oxidative stress, and a variety of literature now reports the impact of oxidative stress on the development of EMT.^[20,154]^ For example, oxidative-antioxidant imbalance in EMT may lead to substantial changes in granulosa cells, including genetic alterations, and adversely affect oocyte maturation.^[155]^ Second, recent studies lie in the role of iron overload-dependent ferroptosis in the pathophysiology of EMT, that is, whether EMT can be treated by selectively inducing ferroptosis. We have discussed that many ferroptosis -related pathways, including iron transporter proteins, GSH/GPX and mevalonate have emerged as potential targets for therapy. In this review we summarize the following. First, the presence of excessive iron concentrations in EMT patients induces oxidative stress and lipid peroxidation. Second, ferroptosis as a death pathway manifests as cellular damage caused by lipid peroxidation, and endometriotic cells increase cellular resistance to ferroptosis through multiple pathways to implant and proliferate in the peritoneal cavity. Third, the use of appropriate antioxidants in the study resisted the toxic effects of reactive oxygen species generated by iron metabolism.

In summary, the molecular mechanisms and signaling pathways involved in ferroptosis are closely related to the development of EMT. Therapies targeting the ferroptosis pathway hold great promise for the future. However, long-term and adequate clinical trials are needed to elucidate the molecular and cellular mechanisms of oxidative stress in EMT and to find better bioactive compounds to improve oxidative stress, chronic pelvic pain and dysmenorrhea.

## Author contributions

**Conceptualization:** Chenghong Ni.

**Writing – original draft:** Chenghong Ni.

**Writing – review & editing:** Chenghong Ni, Dingheng Li.
